# ACEs family genes: Important molecular links between lung cancer and COVID‐19

**DOI:** 10.1002/ctm2.615

**Published:** 2021-12-15

**Authors:** Yinjiang Zhang, Lu Fan, Rongfei Yao, Xu He, Linyi Zhao, Binan Lu, Zongran Pang

**Affiliations:** ^1^ School of Pharmacy Minzu University of China Beijing P. R. China; ^2^ Key Laboratory of Ethnomedicine (Minzu University of China) Ministry of Education Beijing P.R. China

Dear Editor,

COVID‐19 associated with SARS‐CoV‐2 virus is an on‐going global pandemic.^1^ Although great efforts have been made, the COVID‐19 situation is still very serious due to the rapid mutation rate of SARS‐COV‐2 and the increase of drug resistance.^2^ Patients with lung cancer are more susceptible to COVID‐19 because of their immunosuppressed state and fragile lung tissue.^3^, ^4^ angiotensin converting enzyme 2 (*ACE2*) has been confirmed to be the key entry site for the SARS‐CoV‐2 virus^5^; however, the roles of *ACE* and *TMEM27*, the other two genes in ACEs gene family (ACEs) with high homology to *ACE2*, in lung cancer and COVID‐19 have not been entirely clarified.

As shown in Figure 1, data related to ACEs, SARS‐CoV‐2 and lung cancer were obtained from several databases. The relationship among the three was analyzed to provide ideas for prevention and control of SARS‐CoV‐2 infection of lung cancer patients. The transcriptional levels of ACEs in 20 cancers were compared to transcription profiles in normal tissues. The dates showed that the transcriptional level of *ACE* was downregulated in four studies, whereas transcriptional levels of *ACE2* and *TMEM27* were only one study elevated in patients with lung cancer (Figure S1, Table S1). And we further revalidated the transcription level of ACEs in lung cancer patients in UALCAN database. It was found that *ACE* was dramatically downregulated in lung tumor tissues (Figure S2A,B), whereas *ACE2* was at a high transcriptional level in lung tumor tissues (Figure S2C,D). *TMEM27* was overexpressed in lung adenocarcinoma (LUAD), while it was downregulated in lung squamous carcinoma (LUSC) (Figure S2E,F). We further checked the protein expression levels of ACEs in lung cancer (Figure S3A‐C).

**FIGURE 1:**
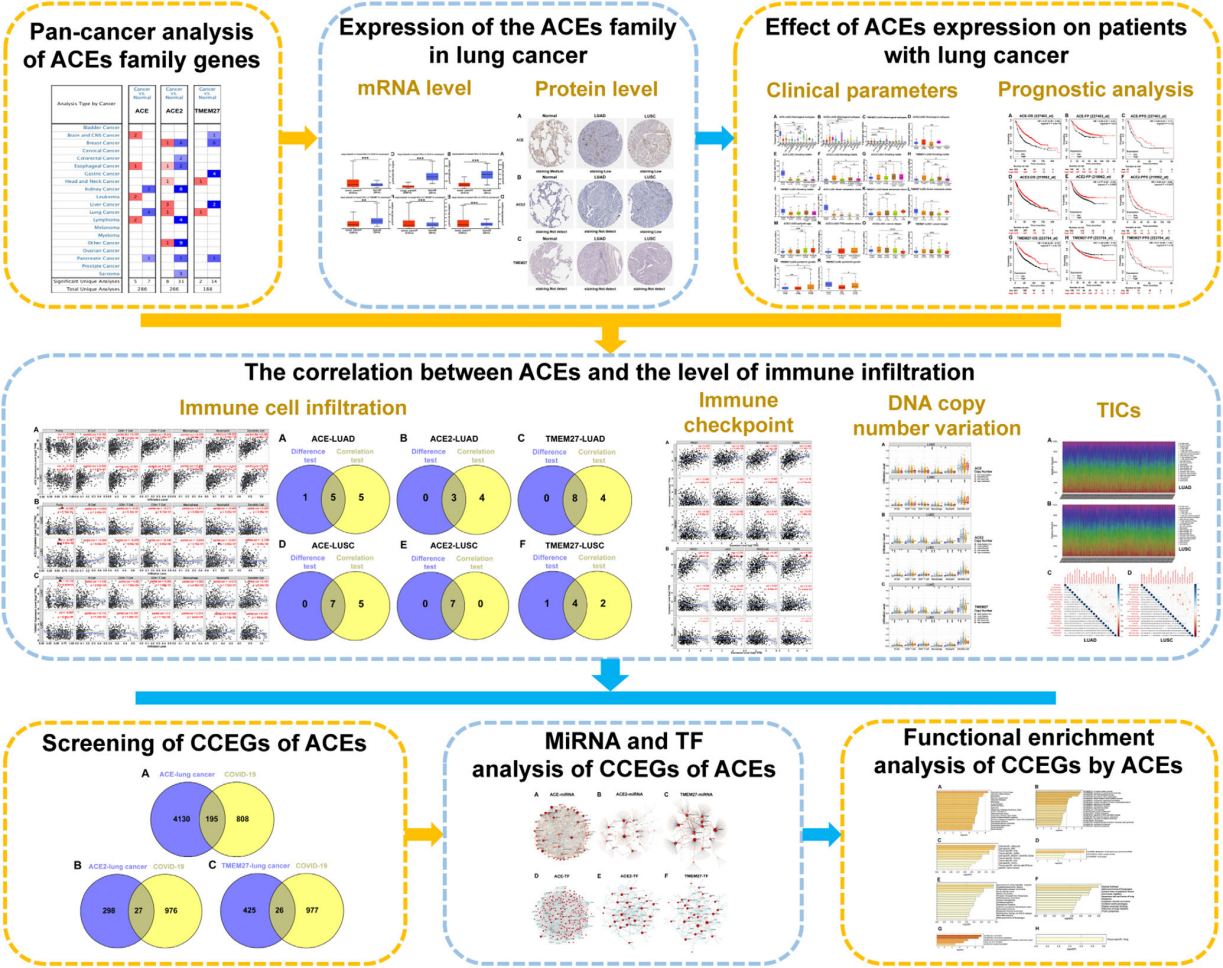
A flow chart showing the overall analysis process of the study. CCEGs: commonly co‐expression genes of ACEs in lung cancer and COVID‐19. ACEs: *ACE*, *ACE2*, *TMEM27*

Then, the relationship between transcriptional levels of ACEs and the clinicopathological parameters of lung cancer patients was investigated. As shown in Figure S4, the results showed that *ACE* was significantly differentially expressed in histological subtypes of LUAD patients, as well as nodal metastasis and smoking habits of LUSC patients. *ACE2* expression was not only significantly different in age, TP53 mutation status, histological subtypes and smoking habits of LUAD, but also in the histological subtypes, smoking habits and individual cancer stage of LUSC patients. *TMEM27* was significantly differentially expressed in gender, nodal metastasis status and smoking habit of LUSC patients, as well as gender, nodal metastasis status, smoking habit, individual cancer stage and histological subtype of LUAD patients. Figure S5 reveals that the prognosis of patients with lung cancer was remarkably influenced by the expression levels of mRNA of most ACEs family members. Specifically, high expression levels of mRNAs of *ACE*, *ACE2* and *TMEM27* predicted better overall survival of lung cancers patients. Higher mRNA expression of ACE and *ACE2* correlated with good first progression of lung cancers patients. Upregulation of *ACE2* correlated with good post‐progression survival of lung cancers patients. In the independent prognostic analysis for ACEs, only *ACE2* showed the potential to independently influence the prognosis of lung cancer patients (Table S2 **–** S4).

A comprehensive analysis was conducted to assess the association of ACEs with immune cell infiltration. The results showed that *ACE* has a positive correlation with a total of six kinds of immune cells in lung cancer tissues. *ACE2* was only positively related to three immune cells in LUAD tissues. *TMEM27* was positively correlated with infiltration level of the six immune cells in LUSC, and negatively connected to Neutrophil cells in LUAD tissues (Figure 2). Analysis of copy number variations showed that ACEs regulated the infiltration level of immune cells (Figure S6). Further examinations revealed that *ACE* was positively correlated with PDCD1, LAG3, PDCD1LG2 and CD274 immune checkpoints both in LUAD and LUSC patients. Notably, *ACE2* showed a negative correlation with PDCD1, LAG3, PDCD1LG2 and CD274 in LUAD patients, but a positive correlation with CD274 in LUSC patients (Figure 3). Multivariate COX survival analysis revealed that Stage2, Stage3, Stage4 and B cells were independent factors that predict the prognosis of patients with LUAD (Table S5), whereas age, stage3, *ACE* and *ACE2* could independently predict the prognosis of patients with LUSC (Table S6).

**FIGURE 2:**
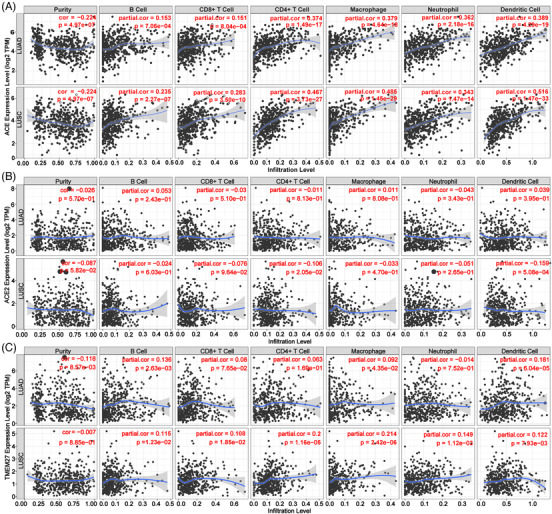
Association of ACEs transcriptional levels with immune infiltration level in lung cancer (TIMER database). (A) *ACE* expression was startlingly positively correlated with six immune cells in lung tumor. (B) *ACE2* was positively related to three immune cells only in LUAD patients. (C) *TMEM27* only showed a negative correlation with Neutrophil cells in LUAD. Filter criteria: *p*‐value < 0.05

**FIGURE 3:**
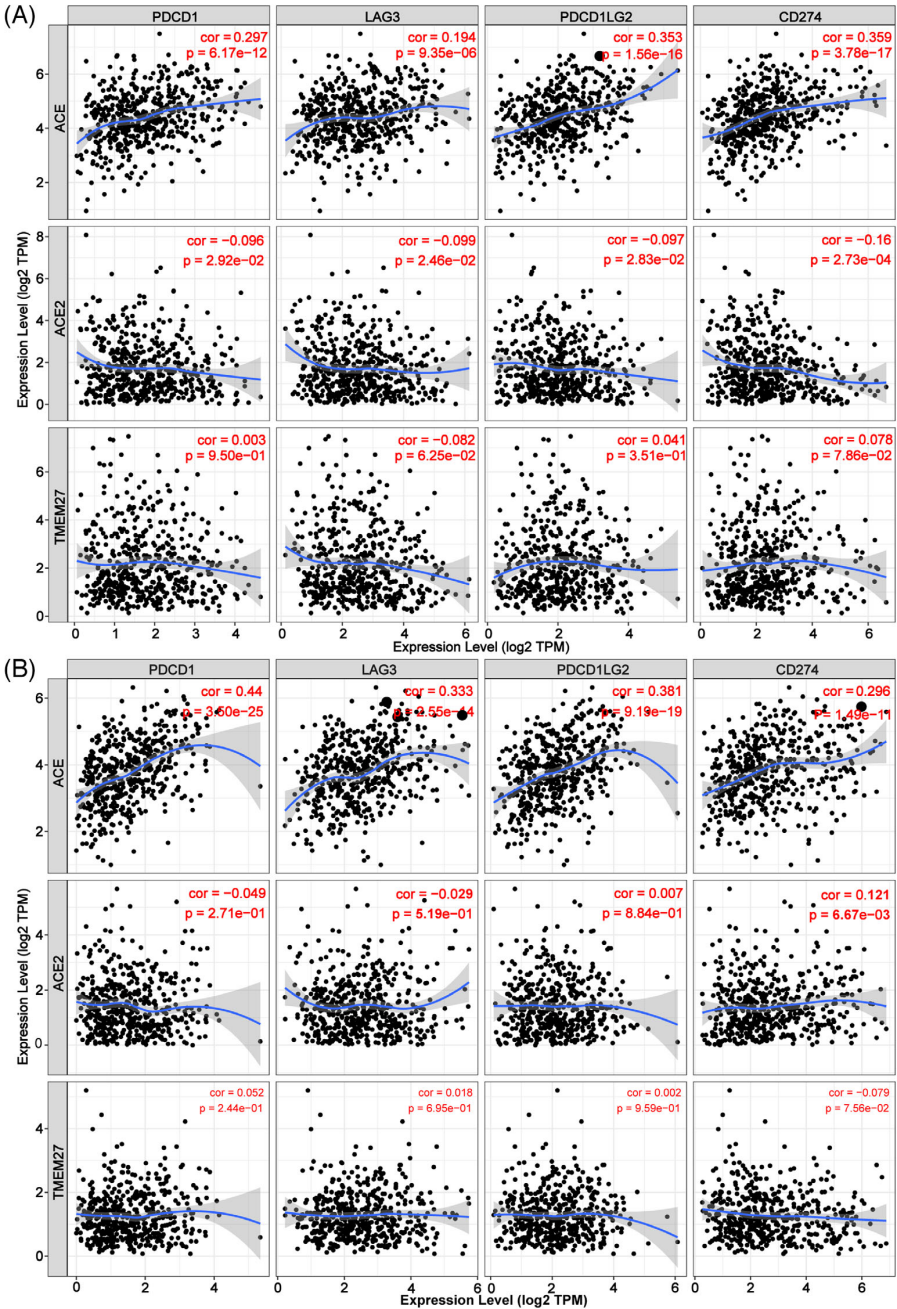
Relevance of ACEs transcriptional levels with immune checkpoints in lung cancer (TIMER database). (A) *ACE* was positively, whereas *ACE2* was negatively connected to PDCD1, LAG3, PDCD1LG2 and CD274 in LUAD. (B) *ACE* was positively related to PDCD1, LAG3, PDCD1LG2 and CD274, *ACE2* showed a positive correlation with CD274 in LUSC. Filter criteria: *p*‐value < 0.05

The relevance of ACEs expression with immune status was further ascertained by CIBERSORT algorithm^6^; 22 types of tumor‐infiltrating immune cells in lung cancer samples were determined (Figure S7). Figures S8–S9 displayed a violin diagram showing immune cell infiltration levels in lung cancer samples from the ACEs high and low expression group. The intersection results of different analyses and correlation analyses were obtained as shown in Figure S10. Results demonstrated that ACEs expression level was strongly related to the infiltration of immune cells.

Finally, a total of 1003 differentially expressed genes (DEGs) in COVID‐19 were identified (Figure S11). Moreover, co‐expressed genes in lung cancer were identified, including 4325 genes of *ACE*, 325 genes of *ACE2* and 451 genes of *TMEM27*. The overlapping area between the co‐expressed genes of ACEs in lung cancer and the DEGs of COVID‐19 was examined. Overall, 195 (*ACE*), 27 (*ACE2*) and 26 (*TMEM27*) genes were selected as the commonly co‐expressed genes (CCEGs) of ACEs in lung cancer patients with COVID‐19. Subsequently, the miRNAs and transcription factors networks targeting CCEGs were constructed (Figure S12). In addition, functional enrichment analysis of CCEGs of ACEs was carried out as shown in Figure 4. The results implied that the CCEGs were largely enriched in circulatory system process and pigment P450 metabolism. Moreover, CCEGs were enriched in lung, liver and spleen tissues. Table S7 illustrates that ACEs were correlated with COVID‐19‐related gene sets. Multiple drugs that target CCEGs of ACEs were predicted by enricher database (Table S8). Acid red, tetradioxin and etoposide were identified as the drugs with the most significant effect on CCEGs of *ACE*, *ACE2*, *TMEM27*, respectively.

**FIGURE 4:**
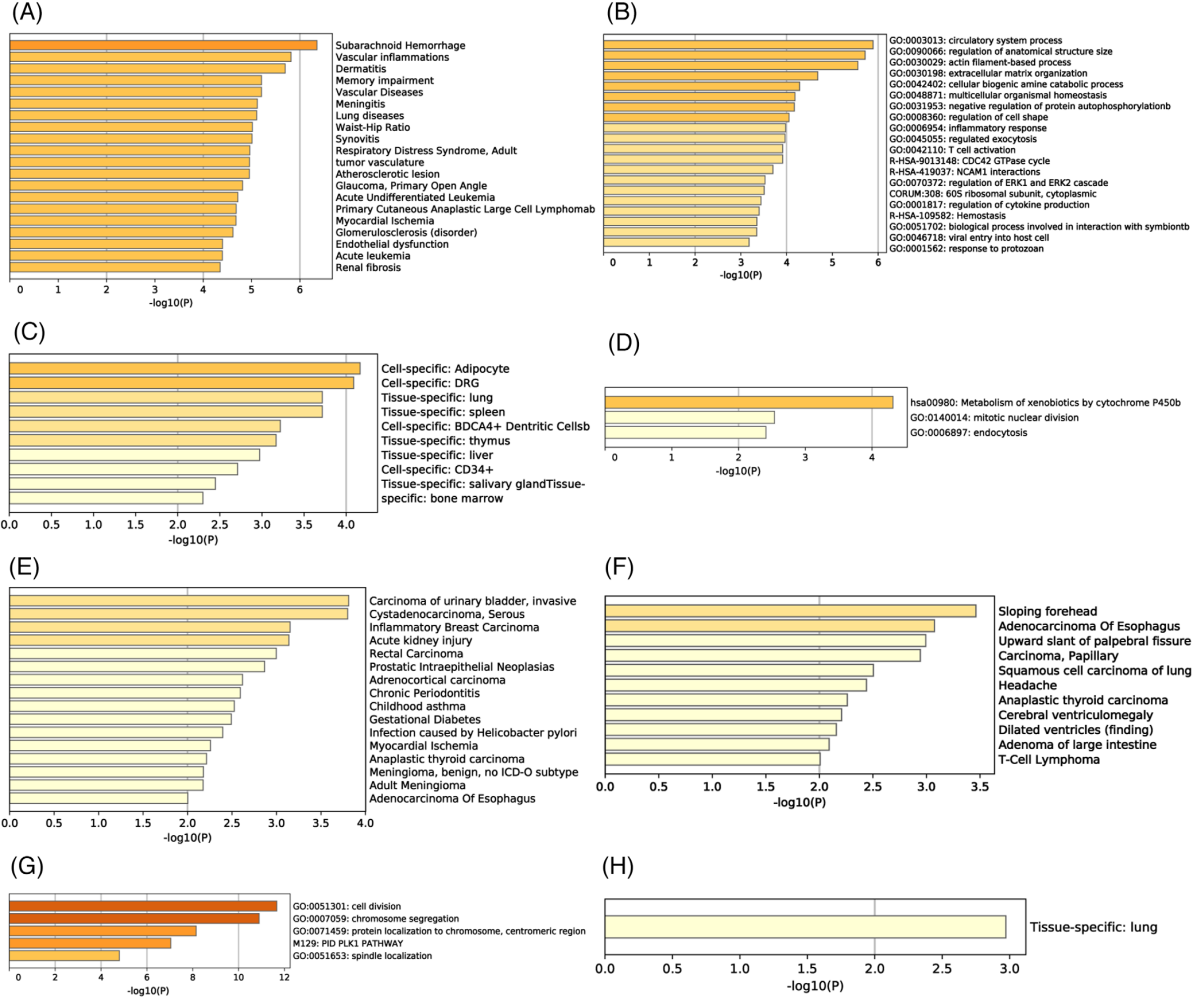
Functional enrichment analysis of commonly co‐expressed genes (CCEGs) on the Metascape database. The gene ontology and kyoto encyclopedia of genes and genomes analyses for CCEGs of *ACE* (B), *ACE2* (D) and *TMEM27*(G). PaGenBase analysis of CCEGs of *ACE* (C) and *TMEM27*(H). DisGeNET analysis of CCEGs of *ACE* (A), *ACE2* (E) and *TMEM27* (F)

In conclusion, this study shows that ACEs may directly or indirectly affect the development and progression of COVID‐19 in lung cancer patients. This finding can be leveraged to develop effective prevention and treatment strategies for lung cancer patients with COVID‐19 infection.

## CONFLICT OF INTEREST

All authors declare that no competing interest exists.

## Supporting information

Supporting Information
