# Supplementary material for: ACEs family genes: Important molecular links between lung cancer and COVID‐19
Source: Clin Transl Med. 2021 Dec 15;11(12):e615. doi: 10.1002/ctm2.615 (PMC8673100; doi:10.1002/ctm2.615)
Supplement: Supplementary file 1 — Supporting Information [file CTM2-11-e615-s001.zip › Supplementary material/Supplementary material.docx]

**SUPPLEMENTARY MATERIAL**

**Figure S1.** **Transcription level of** **ACEs in 20 different types of cancer diseases in the ONCOMINE database.** Differences in the transcription of ACEs were compared with the students’ t-test. Cut-off of *p*-value: 0.01. fold change: 1.5, gene rank: 10%, data type: mRNA.

**Figure S2. The mRNA expression of ACEs in two types of lung tumor and normal lung tissues obtained from UALCAN database**. Expression level of *ACE* in LUAD **(A)**, LUSC **(B)** and the corresponding normal lung tissues. *ACE2* expression level in LUAD **(C)**, LUSC **(D)** and the corresponding normal lung tissues. TMEM27 expression level in LUAD (**E**) and LUSC (**F**) compared to normal lung tissues. * indicates P<0.05, ** indicates P<0.01, *** indicates P<0.001. filter criteria: p-value: 0.01, fold change: 1.5, gene rank: 10%. Abbreviation: LUAD: Lung adenocarcinoma; LUSC: lung squamous carcinoma.

**Figure S3. Representative immunohistochemical images of different ACEs family members in LUAD, LUSC tissues and normal liver tissues screened from the Human Protein Atlas database. (A)** *ACE* protein was expressed at an intermediate level in normal lung tissues, but at a low level in LUAD and LUSC tissues. **(B)** *ACE*2 protein was not detected in LUAD and normal lung tissues, however, it showed low expression in LUSC tissues. **(C)** *TMEM27* protein was detected in LUAD, LUSC and normal lung tissues. Abbreviation: LUAD: Lung adenocarcinoma, LUSC: lung squamous carcinoma.

**Figure S4. Association of mRNA levels of different ACEs family members with the tumor clinical parameters in patients with LUAD and LUSC (UALCAN database).** *ACE* was associated with histological subtypes **(A)** of LUAD patients, as well as smoking habits **(E)** and nodal metastasis status **(J)** of LUSC patients. *ACE2* correlated with histological subtypes **(B)**, smoking habits **(F)**, age **(M)** and TP53 mutation status **(N)** of LUAD, as well as histological subtypes **(D)**, smoking habits **(G)**, and individual cancer stage **(O)** of LUSC patients. *TMEM27* showed strong correlation with histological subtypes **(C)**, smoking habit **(H)**, nodal metastasis status **(K)**, individual cancer stage **(P)** and gender (**Q**) of LUAD patients. In LUSC patients, TMEM27 was associated with smoking habits (**I**), nodal metastasis status (**L**) and gender (**R**). * indicates P<0.05, ** indicates P<0.01, *** indicates P<0.001. Comparative methods: student’s-test, filter criteria: p-value <0.05. Abbreviation: LUAD: Lung adenocarcinoma; LUSC: lung squamous carcinoma.

**Figure S5. Effect of differential expression of ACEs family members on the survival of patients with lung cancer (Kaplan-Meier plotter). (A-C)** The effect of *ACE* mRNA expression on the OS, RFS and PPS of lung cancer patients. (**D-F**) The impact of ACE2 mRNA expression on the OS, FP and PPS of lung cancer patients. (**G-I**) Association of of TMEM27 mRNA expression with the OS, RFS and PPS of lung cancer patients. The red color indicates high expression and black color indicates low expression. filter criteria: p-value <0.05. Abbreviation: LUAD: Lung adenocarcinoma; LUSC: lung squamous carcinoma. OS: overall survival, FP: first progression, PPS: post-progression survival.

**Figure S6. Association of copy number variation of ACEs with immune infiltration level in LUAD and LUSC patients (TIMER database).** The correlation between the abundance of immune infiltrates and copy number variation of *ACE* **(A)**, *ACE2* **(B)**, *TMEM27* **(C)**. The copy number variation includes deep deletion, shallow deletion, diploid/normal, low-level gain, and high amplification. filter criteria: *p*-value <0.05. Abbreviation: LUAD: Lung adenocarcinoma; LUSC: lung squamous carcinoma.

**Figure S7. TIC profile of tumor samples (TCGA database).** A Bar plot showing the proportion of 21 types of TICs in LUAD **(A)** and LUSC **(B)** tumor samples. Column names of the plot represent the sample ID. A heatmap (**C, D**) showing the correlation between 21 types of TICs. Numerical numbers shown in each tiny box indicate the p-value of correlation between two types of cells. The shade of each tiny color box represents the corresponding correlation value between two cells. filter criteria: Pearson correlation coefficient＞0.3, p-value＜0.05. Abbreviation: LUAD: Lung adenocarcinoma; LUSC: lung squamous carcinoma. TICs: tumor-infiltrating immune cells.

**Figure S8. The relationship of ACEs expression with expression levels of** 21 **immune cells in LUAD tumor samples (TCGA database).** Wilcoxon rank-sum test was used to compare differential abundance of immune cells between low and high expression groups of *ACE* **(A),** *ACE2* **(B)**, *TMEM27* (**C**) in LUAD tumor samples. filter criteria: p-value＜0.05. Abbreviation: LUAD: Lung adenocarcinoma.

**Figure S9 Association of ACEs levels on expression level of 21 immune cells in LUSC tumor samples (TCGA database).** The Wilcoxon rank-sum test was used to compare the differential abundance of immune cells between low and high expression groups of ACE (**A**), ACE2 (**B**), TMEM27 (**C**) in LUSC tumor samples. filter criteria: p-value＜0.05. Abbreviation: LUSC: lung squamous carcinoma.

**Figure S10. Correlation between TICs and ACEs expression analyzed by difference studies and correlation studies in LUAD and LUSC (Venny database). (A-C)** *ACE*, *ACE2*, *TMEM27* related TICs were 5, 3, and 8 in LUAD, respectively. **(D-F)** *ACE*, *ACE2*, *TMEM27* related TICs were 7, 7, and 4 in LUSC, respectively. Abbreviation: LUAD: Lung adenocarcinoma; LUSC: lung squamous carcinoma. TICs: tumor-infiltrating immune cells.

**Figure S11. Identification of common genes co-expressed (CCEGs) in ACEs, lung cancer and COVID-19 (Venny database).** A total of 195, 27, and 26 CCEGs of *ACE* **(A)**, *ACE2* **(B)** and *TMEM27* **(C)** were identified. Abbreviation: LUAD: Lung adenocarcinoma; LUSC: lung squamous carcinoma.

**Figure S12. Construction of interaction maps for CCEGs of ACEs and the corresponding miRNA and transcription factor (TF) networks (Networkanalyst database). (A-C)** Interaction network between miRNA of target genes and CCEGs of *ACE*, *ACE2* and *TMEM27*. **(D-F)** Interaction network between TF of target genes and CCEGs of *ACE*, *ACE2* and *TMEM27*. The red circle represents CCEGs, the orange circle indicates miRNA, and blue squares represent TF. Abbreviation: CCEGs: commonly co-expression genes.

**Table S1. Significant changes of ACEs expression in transcription level between lung cancer and normal lung tissues.**

**Table S2. Multivariate analysis.**

**Table S3. Multivariate analysis.**

**Table S4. Multivariate analysis.**

**Table S5. Independent prognostic analysis of ACEs gene family members in LUAD.**

**Table S6. Independent prognostic analysis of ACEs gene family members in LUSC.**

**Table S7. Enrichment analysis of CCEGs of ACEs gene family members in COVID-19 related gene sets.**

**Table S8. Drugs enriched with CCEGs of ACEs gene family members.**
