# Supplementary material for: ACEs family genes: Important molecular links between lung cancer and COVID‐19
Source: Clin Transl Med. 2021 Dec 15;11(12):e615. doi: 10.1002/ctm2.615 (PMC8673100; doi:10.1002/ctm2.615)
Supplement: Supplementary file 1 — Supporting Information [file CTM2-11-e615-s001.zip › Supplementary material/Supplementary material-Tables/Table S1.docx]

| **Table S1. Significant changes of ACEs expression in transcription level between lung cancer and normal lung tissues** | | | | |
| --- | --- | --- | --- | --- |
| **Gene** | **Types of cancer VS. Nomal** | **Fold Change** | **P value** | **PMID** |
| ACE | Lung Adenocarcinoma（n=127） vs. Normal（n=17） | -6.895 | 1.88E-07 | PMID: 11707567 |
|  | Squamous Cell Lung Carcinoma（n=21）vs. Normal（n=17） | -4.914 | 9.66E-04 | PMID: 11707567 |
|  | Lung Carcinoid Tumor（n=20） vs. Normal（n=17） | -5.682 | 1.23E-06 | PMID: 11707567 |
|  | Lung Adenocarcinoma （n=20）vs. Normal（n=19） | -3.313 | 2.63E-06 | PMID: 16314486 |
| ACE2 | Lung Adenocarcinoma（n=226） vs. Normal（n=20） | 2.039 | 1.36E-11 | PMID: 22080568 |
| TMEM27 | Lung Adenocarcinoma（n=226） vs. Normal（n=20） | 2.512 | 8.49E-14 | PMID: 22080568 |

N=Number of samples，“P<0.05” indicates a significant difference.
