# Supplementary material for: ACEs family genes: Important molecular links between lung cancer and COVID‐19
Source: Clin Transl Med. 2021 Dec 15;11(12):e615. doi: 10.1002/ctm2.615 (PMC8673100; doi:10.1002/ctm2.615)
Supplement: Supplementary file 1 — Supporting Information [file CTM2-11-e615-s001.zip › Supplementary material/Supplementary material-Tables/Table S2.docx]

| **Table S2. Multivariate analysis** | | |
| --- | --- | --- |
|  | **P value** | **Hazard Ratio** |
| Gender | 0.0422 | 1.76 (1.02 - 3.03) |
| Histology | 0.5983 | 0.87 (0.52 - 1.46) |
| Stage | 0.1823 | 3.16 (0.58 - 17.17) |
| AJCC stage T | 0.0215 | 2.18 (1.12 - 4.24) |
| AJCC stage N | 0.4983 | 0.56 (0.1 - 3.02) |
| Smoking history | 0.9387 | 1.03 (0.47 - 2.27) |
| ACE | 0.1587 | 1.45 (0.86 - 2.44) |

“P<0.05” indicates a significant difference.
