# Supplementary material for: ACEs family genes: Important molecular links between lung cancer and COVID‐19
Source: Clin Transl Med. 2021 Dec 15;11(12):e615. doi: 10.1002/ctm2.615 (PMC8673100; doi:10.1002/ctm2.615)
Supplement: Supplementary file 1 — Supporting Information [file CTM2-11-e615-s001.zip › Supplementary material/Supplementary material-Tables/Table S3.docx]

| **Table S3. Multivariate analysis** | | |
| --- | --- | --- |
|  | **P value** | **Hazard Ratio** |
| Histology | 0.6245 | 1.09 (0.76 - 1.57) |
| Stage | 0.2394 | 0.63 (0.29 - 1.36) |
| AJCC stage T | 0.0064 | 1.79 (1.18 - 2.72) |
| AJCC stage N | 0.0183 | 2.49 (1.17 - 5.32) |
| Gender | 0.007 | 1.66 (1.15 - 2.39) |
| Smoking history | 0.776 | 1.1 (0.57 - 2.13) |
| ACE2 | 0.0307 | 0.67 (0.46 - 0.96) |

“P<0.05” indicates a significant difference.
