# Supplementary material for: ACEs family genes: Important molecular links between lung cancer and COVID‐19
Source: Clin Transl Med. 2021 Dec 15;11(12):e615. doi: 10.1002/ctm2.615 (PMC8673100; doi:10.1002/ctm2.615)
Supplement: Supplementary file 1 — Supporting Information [file CTM2-11-e615-s001.zip › Supplementary material/Supplementary material-Tables/Table S4.docx]

| **Table S4. Multivariate analysis** | | |
| --- | --- | --- |
|  | **P value** | **Hazard Ratio** |
| Histology | 0.5663 | 0.86 (0.5 - 1.46) |
| Stage | 0.3002 | 2.47 (0.45 - 13.68) |
| AJCC stage T | 0.021 | 2.21 (1.13 - 4.34) |
| AJCC stage N | 0.7061 | 0.72 (0.13 - 3.93) |
| Gender | 0.038 | 1.79 (1.03 - 3.1) |
| Smoking history | 0.8892 | 0.95 (0.43 - 2.09) |
| TMEM27 | 0.6196 | 1.15 (0.67 - 1.97) |

“P<0.05” indicates a significant difference.
