# Supplementary material for: ACEs family genes: Important molecular links between lung cancer and COVID‐19
Source: Clin Transl Med. 2021 Dec 15;11(12):e615. doi: 10.1002/ctm2.615 (PMC8673100; doi:10.1002/ctm2.615)
Supplement: Supplementary file 1 — Supporting Information [file CTM2-11-e615-s001.zip › Supplementary material/Supplementary material-Tables/Table S5.docx]

| **Table S5. Independent prognostic analysis of ACEs gene family members in LUAD** | | | | | | |
| --- | --- | --- | --- | --- | --- | --- |
|  | **coef** | **HR** | **95%CI_l** | **95%CI_u** | **p.value** | **sig** |
| **Age** | 0.014 | 1.014 | 0.998 | 1.032 | 0.093 |  |
| **gendermale** | -0.125 | 0.883 | 0.628 | 1.24 | 0.472 |  |
| **stage2** | 0.885 | 2.424 | 1.595 | 3.683 | 0 | ******* |
| **stage3** | 1.14 | 3.127 | 2.035 | 4.805 | 0 | ******* |
| **stage4** | 1.309 | 3.703 | 2.011 | 6.819 | 0 | ******* |
| **Purity** | 0.297 | 1.346 | 0.596 | 3.04 | 0.474 |  |
| **B_cell** | -3.415 | 0.033 | 0.002 | 0.505 | 0.014 | ***** |
| **CD8_Tcell** | 0.111 | 1.117 | 0.159 | 7.876 | 0.911 |  |
| **CD4_Tcell** | 2.688 | 14.697 | 0.985 | 219.199 | 0.051 |  |
| **Macrophage** | 0.369 | 1.447 | 0.09 | 23.325 | 0.795 |  |
| **Neutrophil** | -1.135 | 0.321 | 0.006 | 16.529 | 0.572 |  |
| **Dendritic** | -0.062 | 0.94 | 0.228 | 3.87 | 0.932 |  |
| **ACE** | -0.168 | 0.845 | 0.695 | 1.027 | 0.09 |  |
| **ACE2** | -0.008 | 0.992 | 0.872 | 1.128 | 0.905 |  |
| **TMEM27** | -0.023 | 0.977 | 0.862 | 1.107 | 0.714 |  |
| Rsquare= 0.134 (max possible= 9.75e-01 ) | | | |  |  |  |
| Likelihood ratio test p= 8.39e-08 | | | |  |  |  |
| Wald test p= 2.19e-07 | | | |  |  |  |
| Score (logrank) test p= 2.82e-08 | | | |  |  |  |
| 436 patients with 156 dying | | | |  |  |  |
