# Supplementary material for: ACEs family genes: Important molecular links between lung cancer and COVID‐19
Source: Clin Transl Med. 2021 Dec 15;11(12):e615. doi: 10.1002/ctm2.615 (PMC8673100; doi:10.1002/ctm2.615)
Supplement: Supplementary file 1 — Supporting Information [file CTM2-11-e615-s001.zip › Supplementary material/Supplementary material-Tables/Table S6.docx]

| **Table S6. Independent prognostic analysis of ACEs gene family members in LUSC** | | | | | | |
| --- | --- | --- | --- | --- | --- | --- |
|  | **coef** | **HR** | **95%CI_l** | **95%CI_u** | **p.value** | **sig** |
| **Age** | 0.019 | 1.019 | 1.002 | 1.037 | 0.033 | ***** |
| **gendermale** | 0.321 | 1.379 | 0.968 | 1.963 | 0.075 |  |
| **stage2** | 0.108 | 1.114 | 0.787 | 1.576 | 0.542 |  |
| **stage3** | 0.515 | 1.674 | 1.144 | 2.448 | 0.008 | ****** |
| **stage4** | 0.903 | 2.466 | 0.879 | 6.921 | 0.086 |  |
| **Purity** | -0.384 | 0.681 | 0.322 | 1.441 | 0.315 |  |
| **B_cell** | 1.411 | 4.101 | 0.329 | 51.184 | 0.273 |  |
| **CD8_Tcell** | -1.717 | 0.18 | 0.029 | 1.125 | 0.067 |  |
| **CD4_Tcell** | -0.055 | 0.947 | 0.066 | 13.672 | 0.968 |  |
| **Macrophage** | -1.314 | 0.269 | 0.021 | 3.457 | 0.313 |  |
| **Neutrophil** | 1.231 | 3.423 | 0.117 | 99.815 | 0.475 |  |
| **Dendritic** | 0.276 | 1.317 | 0.282 | 6.153 | 0.726 |  |
| **ACE** | 0.195 | 1.216 | 1.003 | 1.473 | 0.047 | ***** |
| **ACE2** | -0.222 | 0.801 | 0.67 | 0.959 | 0.015 | ***** |
| **TMEM27** | 0.208 | 1.231 | 0.976 | 1.552 | 0.08 |  |
| Rsquare= 0.069 (max possible= 9.89e-01 ) | | | |  |  |  |
| Likelihood ratio test p= 5.53e-03 | | | |  |  |  |
| Wald test p= 6.22e-03 | | | |  |  |  |
| Score (logrank) test p= 4.89e-03 | | | |  |  |  |
| 452 patients with 193 dying | | | |  |  |  |
