# Supplementary material for: ACEs family genes: Important molecular links between lung cancer and COVID‐19
Source: Clin Transl Med. 2021 Dec 15;11(12):e615. doi: 10.1002/ctm2.615 (PMC8673100; doi:10.1002/ctm2.615)
Supplement: Supplementary file 1 — Supporting Information [file CTM2-11-e615-s001.zip › Supplementary material/Supplementary material-Tables/Table S7.docx]

| **Table S7. Enrichment analysis of CCEGs of ACEs gene family members in COVID-19 related gene sets** | | | | |
| --- | --- | --- | --- | --- |
|  | **Term** | **Overlap** | **Adjusted P-value** | **Genes** |
| **ACE** | Down-regulated by SARS-CoV-2 in ferret trachea from GSE147507 | 21/434 | 2.69E-07 | SEMA6B;GPSM1;TAGLN;RAMP3;GALNT18;CRIP2;LTBP3;MPO;SLC2A6;NR4A1;DES;COL6A2;ATOH8;CDC42EP2;ITGA8;SERPING1;CDC42EP1;NES;ENG;ADAMTS7;LTF |
|  | SARS perturbation Up Genes airway epithelium (HAE) from GSE47961:GPL6480:3 | 17/286 | 2.69E-07 | SAMD9L;GYPC;ZBTB32;HBA2;PARP14;IFIT2;FRMD3;IL4I1;NR4A1;IRF4;LAMP3;EPSTI1;TEK;SLC15A3;XAF1;JUNB;GBP4 |
|  | SARS perturbation Up Genes airway epithelium (HAE) from GSE47961:GPL6480:4 | 19/388 | 5.01E-07 | SAMD9L;PARP14;IFIT2;TYMP;RASGRP3;IL4I1;NR4A1;LAMP3;EPSTI1;CDC42EP2;SERPING1;TNFRSF8;LONRF3;SLC15A3;XAF1;JUNB;TRIM22;GBP4;ITGA9 |
| **ACE2** | COVID-19 patients BALF up | 5/679 | 0.152333626 | TTC9;PCP4L1;COBLL1;GSTA1;TACSTD2 |
|  | SARS Perturbation Down Genes Mouse Lung from GSE19137:GPL1261:3 | 2/134 | 0.253910848 | SELENBP1;SFTPD |
|  | SARS Perturbation Up Genes Mouse Lung from GSE68820:GPL7202:3 | 3/430 | 0.253910848 | MAB21L3;UBE2C;DSCC1 |
| **TMEM27** | SARS Perturbation Up Genes Mouse Lung from GSE68820:GPL7202:3 | 12/430 | 4.07E-12 | ARHGAP11A;TPX2;ASPM;CDCA2;PRC1;PLK1;PLD4;BUB1B;OIP5;KIF23;SKA3;CENPA |
|  | SARS Perturbation Up Genes Mouse Lung from GSE68820:GPL7202:2 | 12/441 | 4.07E-12 | ARHGAP11A;TPX2;ASPM;CDCA2;PRC1;PLK1;PLD4;BUB1B;OIP5;KIF23;SKA3;CENPA |
|  | SARS-CoV perturbation Up Genes bronchial epithelial 2B4 from GSE17400:GPL570:1 | 7/272 | 1.37E-06 | TPX2;ASPM;NAPSA;CDCA2;PRC1;PLK1;BUB1B |
