# Supplementary material for: ACEs family genes: Important molecular links between lung cancer and COVID‐19
Source: Clin Transl Med. 2021 Dec 15;11(12):e615. doi: 10.1002/ctm2.615 (PMC8673100; doi:10.1002/ctm2.615)
Supplement: Supplementary file 1 — Supporting Information [file CTM2-11-e615-s001.zip › Supplementary material/Supplementary material-Tables/Table S8.docx]

| **Table S8. Drugs enriched with CCEGs of ACEs gene family members** | | | |
| --- | --- | --- | --- |
|  | **Term** | **Overlap** | **Adjusted P-value** |
| ACE | Acid red 87 BOSS | 11/198 | 0.004222814 |
|  | 3'-Azido-3'-deoxythymidine CTD 00007047 | 15/374 | 0.004222814 |
|  | estradiol CTD 00005920 | 68/4336 | 0.009872498 |
|  | methyprylon BOSS | 8/127 | 0.014945616 |
|  | HEMATOXYLIN BOSS | 11/251 | 0.014945616 |
|  |  |  |  |
| ACE2 | Tetradioxin CTD 00006848 | 14/3768 | 0.068175416 |
|  | hydrocortisone CTD 00006117 | 3/97 | 0.068175416 |
|  | 8-Anilino-1-naphthalenesulfonic acid CTD 00001273 | 2/23 | 0.068175416 |
|  | CUMENE HYDROPEROXIDE CTD 00000338 | 2/23 | 0.068175416 |
|  | Rifampicin CTD 00006701 | 3/133 | 0.093523851 |
|  |  |  |  |
| TMEM27 | etoposide MCF7 DOWN | 8/48 | 4.41E-13 |
|  | resveratrol MCF7 DOWN | 8/104 | 1.44E-10 |
|  | ciclopirox MCF7 DOWN | 7/67 | 3.44E-10 |
|  | trifluridine MCF7 DOWN | 6/33 | 3.44E-10 |
|  | LUCANTHONE CTD 00006227 | 9/213 | 3.89E-10 |
